# 2,4-Bis(4-bromo­phen­yl)-3-aza­bicyclo­[3.3.1]nonan-9-one

**DOI:** 10.1107/S1600536809017565

**Published:** 2009-05-20

**Authors:** P. Parthiban, V. Ramkumar, S. Amirthaganesan, Yeon Tae Jeong

**Affiliations:** a Division of Image Science and Information Engineering, Pukyong National University, Busan 608 739, Republic of Korea; b Department of Chemistry, IIT Madras, Chennai, TamilNadu, India

## Abstract

The title compound, C_20_H_19_Br_2_NO, shows a chair–chair conformation for the aza­bicycle with an equatorial disposition of the 4-bromo­phenyl groups [dihedral angle between the aromatic rings = 16.48 (3)°]. In the crystal, a short Br⋯Br contact [3.520 (4) Å] occurs and the structure is further stabilized by N—H⋯O hydrogen bonds and C—H⋯O inter­actions.

## Related literature

For general background to the biological properties of 3-aza­bicyclo­nona­nes, see: Jeyaraman & Avila (1981 ▶); Hardick *et al.* (1996 ▶); Barker *et al.* (2005 ▶). For different conformations for the aza­bicycle, see: Parthiban *et al.* (2008*a* ▶,*b* ▶,*c* ▶,*d* ▶, 2009 ▶); Smith-Verdier *et al.* (1983 ▶); Padegimas & Kovacic (1972 ▶). For ring puckering analysis, see: Cremer & Pople (1975 ▶).
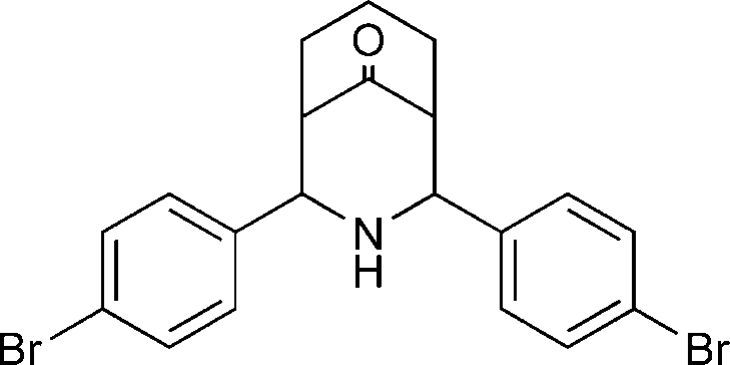


## Experimental

### 

#### Crystal data

C_20_H_19_Br_2_NO
*M* *_r_* = 449.18
Triclinic, 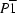

*a* = 6.9415 (3) Å
*b* = 10.4489 (4) Å
*c* = 13.2888 (5) Å
α = 101.542 (2)°
β = 100.391 (2)°
γ = 94.472 (2)°
*V* = 922.34 (6) Å^3^
*Z* = 2
Mo *K*α radiation
μ = 4.40 mm^−1^
*T* = 298 K
0.38 × 0.25 × 0.20 mm

#### Data collection

Bruker SMART APEXII CCD diffractometer
Absorption correction: multi-scan (*SADABS*; Bruker 1999 ▶) *T*_min_ = 0.280, *T*_max_ = 0.415
12376 measured reflections
4036 independent reflections
2805 reflections with *I* > 2σ(*I*)
*R*_int_ = 0.024

#### Refinement

*R*[*F* ^2^ > 2σ(*F* ^2^)] = 0.038
*wR*(*F* ^2^) = 0.089
*S* = 1.02
4036 reflections
221 parameters
H atoms treated by a mixture of independent and constrained refinement
Δρ_max_ = 0.85 e Å^−3^
Δρ_min_ = −0.92 e Å^−3^

### 

Data collection: *SMART* (Bruker–Nonius, 2004 ▶); cell refinement: *SAINT-Plus* (Bruker–Nonius, 2004 ▶); data reduction: *SAINT-Plus* (Bruker–Nonius, 2004 ▶); program(s) used to solve structure: *SHELXS97* (Sheldrick, 2008 ▶); program(s) used to refine structure: *SHELXL97* (Sheldrick, 2008 ▶); molecular graphics: *SHELXTL* (Sheldrick, 2008 ▶); software used to prepare material for publication: *SHELXTL* (Sheldrick, 2008 ▶).

## Supplementary Material

Crystal structure: contains datablocks global, I. DOI: 10.1107/S1600536809017565/hb2967sup1.cif

Structure factors: contains datablocks I. DOI: 10.1107/S1600536809017565/hb2967Isup2.hkl

Additional supplementary materials:  crystallographic information; 3D view; checkCIF report

## Figures and Tables

**Table 1:** Hydrogen-bond geometry (Å, °)

*D*—H⋯*A*	*D*—H	H⋯*A*	*D*⋯*A*	*D*—H⋯*A*
N1—H1*A*⋯O1^i^	0.80 (3)	2.42 (3)	3.191 (3)	162 (3)
C16—H16⋯O1^ii^	0.93	2.53	3.242 (3)	133

Symmetry codes: (i) 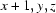; (ii) 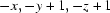.

**Fig. 1.**
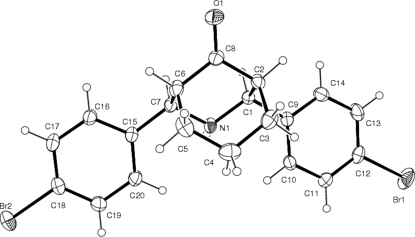
The molecular sturcture of (I) with non-hydrogen atoms represented as 30% probability ellipsoids.

**Fig. 2.**
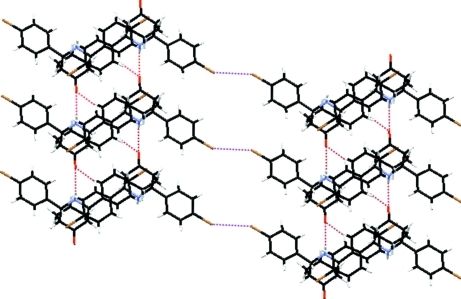
N—H···O interaction and Br—Br interactions (dashed lines) in the crystal of (I).

## supplementary crystallographic information

### Comment

3-Azabicyclononanes are important class of heterocycles due to their broad spectrum of biological activities (Jeyaraman & Avila, 1981; Hardick *et al.*, 1996; Barker *et al.*, 2005). Owing to the diverse possibilities in conformations, *viz*., chair-chair (Parthiban *et al.*, 2008*a*,*b*,*c*,*d*, 2009), chair-boat (Smith-Verdier *et al.*, 1983) and boat-boat (Padegimas & Kovacic, 1972) for the azabicycle, the present crystal study was undertaken to explore the conformation, stereochemistry and bonding of the title compound, (I).

The analysis of torsion angles, asymmetry parameters and least-squares plane calculation of the title compound shows that the piperidine ring adopts near ideal chair conformation with the deviation of ring atoms N1 and C8 from the C1/C2/C6/C7 plane by 0.636 (3) and -0.730 (3) Å. respectively; the q2 and q3 are 0.057 (3)Å and -0.610 (3) Å. The total puckering amplitude, Q_T_ = 0.613 (3)Å and θ = 174.5 (3)°. (Cremer & Pople, 1975).

The cyclohexane ring deviates from the ideal chair conformation by the deviation of ring atoms C8 and C4 from the C2/C3/C5/C6 plane by -0.693 (4)Å and 0.547 (3) Å, respectively. Total puckering amplitude, Q_T_ = 0.546 (3) Å, q2 = 0.109 (4) Å, q3 = -0.535 (4)Å and θ =168.6 (4)° (Cremer & Pople, 1975).

Hence, the title compound C_20_ H_19_ Br_2_ N O, exists in twin-chair conformation with equatorial orientation of 4-bromophenyl groups on the heterocycle and are orientated at an angle of 16.48 (3)° to each other. the torsion angle of C8—C2—C1—C9 and C8—C6—C7—C15 are -177.26 (3) and -178.37 (4) °, respectively.

An interesting feature of the crystal structure is a weak intermolecular Br···Br [3.520 (4) Å; symmetry code: 1 - *x*, 1 - *y*, - *z*] interaction which is shorter than the sum of the van der Waals radius of Br atoms. The crystal structure is further stabilized by N—H···O interaction and C—H···O interaction, where the oxygen atom bonds with both C18 and N1 forming a bifurcated bond (Table 1).

### Experimental

To a warm solution of 0.075 mol ammonium acetate in 50 ml absolute ethanol, 0.1 mol of *para*-bromobenzaldehyde and 0.05 mol of cyclohexanone were added. The mixture was gently warmed on a hot plate with stirring till the yellow color formed during the mixing of the reactants and stirring is continued over night at room temparature. At the end, the white crude azabicyclic ketone was separated by filtration and washed with 1:5 ethanol-ether mixture. Colourless blocks of (I) were recrystallised from ethanol.

### Refinement

The N-bound H atom was located in a difference map and refined isotropically.  Other hydrogen atoms were geometrically placed (C—H = 0.93–0.98Å) and refined as riding with *U*_iso_(H) = 1.2*U*_eq_(C).

### Crystal data

**Table:**

C_20_H_19_Br_2_NO	*Z* = 2
*M**_r_* = 449.18	*F*(000) = 448
Triclinic, *P*1	*D*_x_ = 1.617 Mg m^−^^3^
Hall symbol: -P 1	Mo *K*α radiation, λ = 0.71073 Å
*a* = 6.9415 (3) Å	Cell parameters from 4458 reflections
*b* = 10.4489 (4) Å	θ = 2.3–23.7°
*c* = 13.2888 (5) Å	µ = 4.40 mm^−^^1^
α = 101.542 (2)°	*T* = 298 K
β = 100.391 (2)°	Block, colourless
γ = 94.472 (2)°	0.38 × 0.25 × 0.20 mm
*V* = 922.34 (6) Å^3^	

### Data collection

**Table:**

Bruker SMART CCD diffractometer	4036 independent reflections
Radiation source: fine-focus sealed tube	2805 reflections with *I* > 2σ(*I*)
graphite	*R*_int_ = 0.024
φ and ω scans	θ_max_ = 28.3°, θ_min_ = 1.6°
Absorption correction: multi-scan (*SADABS*; Bruker 1999)	*h* = −9→9
*T*_min_ = 0.280, *T*_max_ = 0.415	*k* = −13→10
12376 measured reflections	*l* = −16→17

### Refinement

**Table:**

Refinement on *F*^2^	Primary atom site location: structure-invariant direct methods
Least-squares matrix: full	Secondary atom site location: difference Fourier map
*R*[*F*^2^ > 2σ(*F*^2^)] = 0.038	Hydrogen site location: inferred from neighbouring sites
*wR*(*F*^2^) = 0.089	H atoms treated by a mixture of independent and constrained refinement
*S* = 1.02	*w* = 1/[σ^2^(*F*_o_^2^) + (0.0266*P*)^2^ + 1.1237*P*] where *P* = (*F*_o_^2^ + 2*F*_c_^2^)/3
4036 reflections	(Δ/σ)_max_ = 0.001
221 parameters	Δρ_max_ = 0.85 e Å^−^^3^
0 restraints	Δρ_min_ = −0.92 e Å^−^^3^

### Special details

**Table:**

Geometry. All e.s.d.'s (except the e.s.d. in the dihedral angle between two l.s. planes)are estimated using the full covariance matrix. The cell e.s.d.'s are takeninto account individually in the estimation of e.s.d.'s in distances, anglesand torsion angles; correlations between e.s.d.'s in cell parameters are onlyused when they are defined by crystal symmetry. An approximate (isotropic)treatment of cell e.s.d.'s is used for estimating e.s.d.'s involving l.s. planes.
Refinement. Refinement of *F*^2^ against ALL reflections. The weighted *R*-factor *wR* and goodness of fit *S* are based on *F*^2^, conventional *R*-factors *R* are based on *F*, with *F* set to zero for negative *F*^2^. The threshold expression of *F*^2^ > σ(*F*^2^) is used only for calculating *R*-factors(gt) *etc*. and is not relevant to the choice of reflections for refinement. *R*-factors based on *F*^2^ are statistically about twice as large as those based on *F*, and *R*- factors based on ALL data will be even larger.

### Fractional atomic coordinates and isotropic or equivalent isotropic displacement parameters (Å^2^)

**Table:**

	*x*	*y*	*z*	*U*_iso_*/*U*_eq_	
Br1	0.51212 (7)	−0.32865 (4)	0.04459 (3)	0.07124 (16)	
Br2	0.76344 (6)	0.92093 (4)	0.40986 (3)	0.06669 (15)	
C1	0.2024 (4)	0.1542 (3)	0.2864 (2)	0.0314 (6)	
H1	0.1988	0.1411	0.3571	0.038*	
C2	−0.0130 (4)	0.1605 (3)	0.2300 (2)	0.0354 (7)	
H2	−0.0943	0.0792	0.2291	0.043*	
C3	−0.0360 (5)	0.1813 (3)	0.1177 (2)	0.0469 (8)	
H3A	0.0242	0.1136	0.0771	0.056*	
H3B	−0.1754	0.1706	0.0864	0.056*	
C4	0.0554 (5)	0.3150 (4)	0.1097 (3)	0.0539 (9)	
H4A	0.1974	0.3158	0.1199	0.065*	
H4B	0.0080	0.3287	0.0399	0.065*	
C5	0.0073 (5)	0.4269 (3)	0.1896 (3)	0.0492 (8)	
H5A	−0.1279	0.4427	0.1669	0.059*	
H5B	0.0921	0.5061	0.1915	0.059*	
C6	0.0320 (4)	0.4019 (3)	0.3006 (2)	0.0381 (7)	
H6	−0.0204	0.4722	0.3446	0.046*	
C7	0.2472 (4)	0.3925 (3)	0.3542 (2)	0.0328 (6)	
H7	0.2449	0.3782	0.4247	0.039*	
C8	−0.0841 (4)	0.2732 (3)	0.2963 (2)	0.0359 (7)	
C9	0.2870 (4)	0.0391 (3)	0.2290 (2)	0.0317 (6)	
C10	0.4256 (4)	0.0528 (3)	0.1678 (2)	0.0365 (7)	
H10	0.4721	0.1363	0.1618	0.044*	
C11	0.4958 (4)	−0.0566 (3)	0.1153 (2)	0.0405 (7)	
H11	0.5920	−0.0464	0.0761	0.049*	
C12	0.4230 (5)	−0.1791 (3)	0.1214 (2)	0.0410 (7)	
C13	0.2850 (5)	−0.1960 (3)	0.1810 (3)	0.0509 (9)	
H13	0.2359	−0.2800	0.1846	0.061*	
C14	0.2202 (5)	−0.0867 (3)	0.2355 (3)	0.0458 (8)	
H14	0.1295	−0.0978	0.2776	0.055*	
C15	0.3781 (4)	0.5192 (3)	0.3658 (2)	0.0324 (6)	
C16	0.3485 (4)	0.6307 (3)	0.4353 (2)	0.0385 (7)	
H16	0.2501	0.6248	0.4739	0.046*	
C17	0.4611 (4)	0.7499 (3)	0.4489 (2)	0.0427 (7)	
H17	0.4379	0.8238	0.4951	0.051*	
C18	0.6087 (4)	0.7574 (3)	0.3925 (2)	0.0402 (7)	
C19	0.6428 (4)	0.6494 (3)	0.3236 (3)	0.0438 (8)	
H19	0.7429	0.6555	0.2862	0.053*	
C20	0.5260 (4)	0.5304 (3)	0.3101 (2)	0.0395 (7)	
H20	0.5480	0.4572	0.2628	0.047*	
N1	0.3217 (3)	0.2793 (2)	0.2964 (2)	0.0328 (5)	
O1	−0.2151 (3)	0.2611 (2)	0.34451 (19)	0.0541 (6)	
H1A	0.434 (5)	0.275 (3)	0.322 (2)	0.039 (9)*	

### Atomic displacement parameters (Å^2^)

**Table:**

	*U*^11^	*U*^22^	*U*^33^	*U*^12^	*U*^13^	*U*^23^
Br1	0.1029 (3)	0.0451 (3)	0.0682 (3)	0.0312 (2)	0.0296 (2)	−0.0015 (2)
Br2	0.0736 (3)	0.0415 (2)	0.0772 (3)	−0.01884 (18)	0.0181 (2)	0.0021 (2)
C1	0.0309 (13)	0.0280 (16)	0.0342 (15)	0.0023 (11)	0.0088 (11)	0.0031 (13)
C2	0.0268 (13)	0.0318 (17)	0.0449 (17)	−0.0002 (11)	0.0073 (12)	0.0034 (14)
C3	0.0399 (17)	0.053 (2)	0.0409 (17)	0.0099 (15)	0.0012 (14)	−0.0005 (16)
C4	0.0465 (18)	0.075 (3)	0.0428 (18)	0.0069 (17)	0.0072 (15)	0.0215 (19)
C5	0.0419 (17)	0.042 (2)	0.061 (2)	0.0033 (14)	−0.0021 (15)	0.0169 (18)
C6	0.0304 (14)	0.0334 (18)	0.0474 (17)	0.0084 (12)	0.0075 (13)	−0.0002 (14)
C7	0.0303 (14)	0.0303 (17)	0.0354 (15)	0.0046 (11)	0.0072 (12)	0.0004 (13)
C8	0.0250 (13)	0.0402 (19)	0.0393 (16)	0.0052 (12)	0.0051 (12)	0.0018 (14)
C9	0.0292 (13)	0.0279 (17)	0.0355 (15)	0.0033 (11)	0.0039 (11)	0.0035 (13)
C10	0.0367 (15)	0.0267 (17)	0.0438 (17)	−0.0008 (12)	0.0095 (13)	0.0032 (14)
C11	0.0420 (16)	0.039 (2)	0.0412 (17)	0.0065 (14)	0.0151 (13)	0.0026 (15)
C12	0.0487 (17)	0.0314 (19)	0.0393 (16)	0.0148 (14)	0.0050 (14)	−0.0009 (14)
C13	0.061 (2)	0.0243 (19)	0.069 (2)	0.0026 (15)	0.0199 (18)	0.0094 (17)
C14	0.0490 (18)	0.035 (2)	0.060 (2)	0.0049 (14)	0.0265 (16)	0.0117 (16)
C15	0.0301 (14)	0.0289 (17)	0.0346 (15)	0.0053 (11)	0.0031 (12)	0.0013 (13)
C16	0.0368 (15)	0.0357 (19)	0.0388 (16)	0.0010 (13)	0.0090 (13)	−0.0019 (14)
C17	0.0474 (17)	0.0328 (19)	0.0413 (17)	0.0047 (14)	0.0062 (14)	−0.0048 (14)
C18	0.0405 (16)	0.0307 (18)	0.0441 (17)	−0.0013 (13)	−0.0001 (13)	0.0062 (15)
C19	0.0384 (16)	0.041 (2)	0.0535 (19)	0.0016 (14)	0.0173 (14)	0.0080 (16)
C20	0.0381 (15)	0.0325 (18)	0.0466 (17)	0.0059 (13)	0.0133 (13)	0.0005 (14)
N1	0.0245 (12)	0.0277 (15)	0.0420 (14)	0.0035 (10)	0.0055 (10)	−0.0013 (11)
O1	0.0373 (12)	0.0599 (16)	0.0651 (15)	0.0028 (10)	0.0245 (11)	0.0019 (13)

### Geometric parameters (Å, °)

**Table:**

Br1—C12	1.899 (3)	C7—H7	0.9800
Br2—C18	1.896 (3)	C8—O1	1.216 (3)
C1—N1	1.461 (4)	C9—C10	1.384 (4)
C1—C9	1.510 (4)	C9—C14	1.384 (4)
C1—C2	1.560 (4)	C10—C11	1.386 (4)
C1—H1	0.9800	C10—H10	0.9300
C2—C8	1.497 (4)	C11—C12	1.362 (4)
C2—C3	1.532 (4)	C11—H11	0.9300
C2—H2	0.9800	C12—C13	1.372 (5)
C3—C4	1.519 (5)	C13—C14	1.377 (5)
C3—H3A	0.9700	C13—H13	0.9300
C3—H3B	0.9700	C14—H14	0.9300
C4—C5	1.516 (5)	C15—C20	1.381 (4)
C4—H4A	0.9700	C15—C16	1.388 (4)
C4—H4B	0.9700	C16—C17	1.379 (4)
C5—C6	1.531 (5)	C16—H16	0.9300
C5—H5A	0.9700	C17—C18	1.380 (4)
C5—H5B	0.9700	C17—H17	0.9300
C6—C8	1.498 (4)	C18—C19	1.369 (4)
C6—C7	1.554 (4)	C19—C20	1.392 (4)
C6—H6	0.9800	C19—H19	0.9300
C7—N1	1.461 (4)	C20—H20	0.9300
C7—C15	1.511 (4)	N1—H1A	0.80 (3)
			
N1—C1—C9	112.3 (2)	O1—C8—C2	123.9 (3)
N1—C1—C2	109.5 (2)	O1—C8—C6	124.0 (3)
C9—C1—C2	110.5 (2)	C2—C8—C6	112.0 (2)
N1—C1—H1	108.1	C10—C9—C14	118.0 (3)
C9—C1—H1	108.1	C10—C9—C1	123.1 (3)
C2—C1—H1	108.1	C14—C9—C1	118.8 (3)
C8—C2—C3	109.2 (3)	C9—C10—C11	120.7 (3)
C8—C2—C1	105.7 (2)	C9—C10—H10	119.7
C3—C2—C1	115.4 (2)	C11—C10—H10	119.7
C8—C2—H2	108.8	C12—C11—C10	119.6 (3)
C3—C2—H2	108.8	C12—C11—H11	120.2
C1—C2—H2	108.8	C10—C11—H11	120.2
C4—C3—C2	114.2 (3)	C11—C12—C13	121.0 (3)
C4—C3—H3A	108.7	C11—C12—Br1	119.4 (2)
C2—C3—H3A	108.7	C13—C12—Br1	119.6 (2)
C4—C3—H3B	108.7	C12—C13—C14	119.0 (3)
C2—C3—H3B	108.7	C12—C13—H13	120.5
H3A—C3—H3B	107.6	C14—C13—H13	120.5
C5—C4—C3	112.7 (3)	C13—C14—C9	121.5 (3)
C5—C4—H4A	109.1	C13—C14—H14	119.2
C3—C4—H4A	109.1	C9—C14—H14	119.2
C5—C4—H4B	109.1	C20—C15—C16	117.9 (3)
C3—C4—H4B	109.1	C20—C15—C7	123.3 (3)
H4A—C4—H4B	107.8	C16—C15—C7	118.8 (2)
C4—C5—C6	114.0 (3)	C17—C16—C15	121.8 (3)
C4—C5—H5A	108.7	C17—C16—H16	119.1
C6—C5—H5A	108.7	C15—C16—H16	119.1
C4—C5—H5B	108.7	C16—C17—C18	118.8 (3)
C6—C5—H5B	108.7	C16—C17—H17	120.6
H5A—C5—H5B	107.6	C18—C17—H17	120.6
C8—C6—C5	108.9 (3)	C19—C18—C17	121.1 (3)
C8—C6—C7	106.3 (2)	C19—C18—Br2	119.8 (2)
C5—C6—C7	115.2 (2)	C17—C18—Br2	119.1 (2)
C8—C6—H6	108.8	C18—C19—C20	119.3 (3)
C5—C6—H6	108.8	C18—C19—H19	120.4
C7—C6—H6	108.8	C20—C19—H19	120.4
N1—C7—C15	112.1 (2)	C15—C20—C19	121.1 (3)
N1—C7—C6	110.0 (2)	C15—C20—H20	119.4
C15—C7—C6	111.1 (2)	C19—C20—H20	119.4
N1—C7—H7	107.8	C1—N1—C7	113.8 (2)
C15—C7—H7	107.8	C1—N1—H1A	110 (2)
C6—C7—H7	107.8	C7—N1—H1A	111 (2)
			
N1—C1—C2—C8	−58.5 (3)	C1—C9—C10—C11	−178.8 (3)
C9—C1—C2—C8	177.2 (2)	C9—C10—C11—C12	2.1 (5)
N1—C1—C2—C3	62.2 (3)	C10—C11—C12—C13	−1.8 (5)
C9—C1—C2—C3	−62.1 (3)	C10—C11—C12—Br1	177.4 (2)
C8—C2—C3—C4	51.9 (3)	C11—C12—C13—C14	−0.1 (5)
C1—C2—C3—C4	−66.9 (4)	Br1—C12—C13—C14	−179.4 (3)
C2—C3—C4—C5	−45.1 (4)	C12—C13—C14—C9	1.8 (5)
C3—C4—C5—C6	45.8 (4)	C10—C9—C14—C13	−1.5 (5)
C4—C5—C6—C8	−53.2 (3)	C1—C9—C14—C13	176.9 (3)
C4—C5—C6—C7	66.0 (4)	N1—C7—C15—C20	12.5 (4)
C8—C6—C7—N1	56.8 (3)	C6—C7—C15—C20	−111.0 (3)
C5—C6—C7—N1	−63.8 (3)	N1—C7—C15—C16	−167.8 (3)
C8—C6—C7—C15	−178.4 (2)	C6—C7—C15—C16	68.6 (3)
C5—C6—C7—C15	61.0 (3)	C20—C15—C16—C17	0.4 (4)
C3—C2—C8—O1	122.1 (3)	C7—C15—C16—C17	−179.2 (3)
C1—C2—C8—O1	−113.2 (3)	C15—C16—C17—C18	−0.9 (5)
C3—C2—C8—C6	−60.8 (3)	C16—C17—C18—C19	0.6 (5)
C1—C2—C8—C6	63.9 (3)	C16—C17—C18—Br2	179.6 (2)
C5—C6—C8—O1	−121.4 (3)	C17—C18—C19—C20	0.1 (5)
C7—C6—C8—O1	113.9 (3)	Br2—C18—C19—C20	−178.9 (2)
C5—C6—C8—C2	61.4 (3)	C16—C15—C20—C19	0.4 (4)
C7—C6—C8—C2	−63.2 (3)	C7—C15—C20—C19	180.0 (3)
N1—C1—C9—C10	−17.2 (4)	C18—C19—C20—C15	−0.6 (5)
C2—C1—C9—C10	105.4 (3)	C9—C1—N1—C7	−178.3 (2)
N1—C1—C9—C14	164.3 (3)	C2—C1—N1—C7	58.5 (3)
C2—C1—C9—C14	−73.0 (3)	C15—C7—N1—C1	178.2 (2)
C14—C9—C10—C11	−0.4 (4)	C6—C7—N1—C1	−57.6 (3)

### Hydrogen-bond geometry (Å, °)

**Table:**

*D*—H···*A*	*D*—H	H···*A*	*D*···*A*	*D*—H···*A*
N1—H1A···O1^i^	0.80 (3)	2.42 (3)	3.191 (3)	162 (3)
C16—H16···O1^ii^	0.93	2.53	3.242 (3)	133

Symmetry codes: (i) *x*+1, *y*, *z*; (ii) −*x*, −*y*+1, −*z*+1.
